# Shoulder girdle neoplasm misdiagnosis and clinical manifestations: A scoping review

**DOI:** 10.1177/17585732251324656

**Published:** 2025-03-13

**Authors:** Michael S Wilkinson, Steven J Obst

**Affiliations:** 1 273488 School of Health, Medical, and Applied Sciences, Central Queensland University, Cairns, QLD, Australia; 2 273488 School of Health, Medical, and Applied Sciences, Central Queensland University, Bundaberg, QLD, Australia

**Keywords:** Frozen shoulder, clinical features, misdiagnosis, shoulder neoplasm

## Abstract

**Introduction:**

The aim of this review is to outline the clinical presentation of patients with shoulder girdle neoplasm to help differentiate it from frozen shoulder contracture syndrome (FSCS) as well as quantify misdiagnosis rates in the literature.

**Methods:**

Four electronic databases were searched (Embase, Medline, PUBMED and Scopus) for cohort studies of patients with shoulder girdle neoplasm with or without misdiagnosis as FSCS in line with the PRISMA-ScR guidelines for scoping reviews.

**Results:**

The initial search yielded 2462 studies, 10 of which were included in the final review. The most common symptom of patients with shoulder girdle neoplasm was shoulder pain (62%) followed by swelling/mass/deformity (34%) and local/bony tenderness (13%). In patients with neoplasm initially misdiagnosed as FSCS the main complaints were shoulder pain and subjective stiffness/loss of range of motion (73% each). Misdiagnosis rates ranged from 10% to 50% and resulted in diagnostic delays of up to 30 months.

**Conclusions:**

Key clinical features of bone and soft tissue tumours such as local bony tenderness and careful observation for swelling/mass/deformity should be included in the physical examination to help differentiate between the two pathologies and help guide the choice of initial imaging for the primary contact clinician.

## Background

Neoplasms of the shoulder girdle include bone tumours of the proximal humerus, scapula, and clavicle and tumours of the surrounding soft tissues.^1^ Soft tissue tumours can occur within the deltoid, rotator cuff, periscapular muscles, brachial plexus and lymph in the upper shoulder girdle, with sarcomas being the third most commonly diagnosed neoplasm in this region.^1,2^ In contrast, non-metastatic bone tumours of the shoulder girdle are relatively rare, but have been reported to account for 10%–15% of highly malignant neoplasms and include osteosarcoma, chondrosarcoma and Ewing's sarcoma.^1,3^ As such, prompt diagnosis and management are essential in patients with these lesions to avoid expansion and metastasis.^4^ One of the primary reasons patients experience delay in the diagnosis of shoulder girdle neoplasm is the similar clinical presentation to other musculoskeletal pathologies.^5,6^ Misdiagnosis and lack of appropriate imaging for neoplasms such as soft tissue sarcoma have been reported to account for mean diagnostic delays of up to 6 months.^5^ Similarly, highly malignant bone tumours have been documented to have diagnostic delays of up to 10.5 months when presenting in the upper limb.^7^ Addressing diagnostic delay due to misdiagnosis is vital as survival prognostics for neoplasms such as Ewing's sarcoma and chondrosarcoma are dependent on time-sensitive variables such as metastasis, tumour size and grading.^8,9^ This emphasises the need for clear clinical decision-making relating to appropriate imaging and onward referral by primary contact clinicians (e.g. general practitioners, physiotherapists etc.).

Shoulder neoplasm, along with other conditions such as glenohumeral osteoarthritis (OA), calcific rotator cuff tendinopathy, posterior shoulder dislocation and frozen shoulder contracture syndrome (FSCS) can all present with patient-reported glenohumeral joint pain and stiffness and/or clinician determined loss of passive range of motion (ROM).^10,11^ The most well-documented of these instances is the reported misdiagnosis of shoulder girdle neoplasm as FSCS.^6,12^ FSCS is a pathology that initially presents with progressing shoulder pain followed by distinct phases of progressive, static, and regressive loss of glenohumeral active and passive ROM.^10,13^ FSCS has a prevalence of up to 5% in the general population and most commonly presents in people aged between 40 and 60 years.^14,15^ This also coincides with the average age at diagnosis for both bone cancer (fifth decade) and soft tissue sarcomas (sixth decade).^16,17^ FSCS is often considered a diagnosis of exclusion through normal shoulder radiographs in the presence of passive glenohumeral joint ROM loss about the affected shoulder.^10,18^ However, there exists debate in the diagnostic pathway of FSCS. Some sources suggest all suspected cases should receive plain radiography to rule out masquerading pathologies, including neoplasm.^4,10,11^ Others suggest this approach offers little diagnostic utility over concise clinical history and examination.^19^ It has also been reported that up to 10% of shoulder girdle neoplasms may not be detected by plain radiography^20^ further emphasising the need for decisive clinical reasoning when determining imaging pathway.

While FSCS typically has a natural course of 1–3 years, some patients can have ongoing pain and disability which exceeds this timeframe.^13^ This protracted period of symptoms during which FSCS takes its natural course can leave those who have been misdiagnosed with this condition without appropriate intervention resulting in diagnostic delays of up to 19 months.^6^ Additionally, many of the interventions for recalcitrant FSCS, such as arthroscopic capsular release and arthrographic distension (hydrodilatation), are absolute contraindications in the presence of any local oncological process.^6^ It is therefore critical that primary contact clinicians are cognizant of the key clinical features that distinguish shoulder neoplasms and FSCS to ensure early and accurate diagnosis and avoid inappropriate interventions and iatrogenic harm.

Given the overlap in symptoms and relative age of onset between shoulder girdle neoplasm and FSCS, it is important to distinguish what clinical features separate each and would necessitate specific or specialised imaging and histopathological testing. Therefore, the aims of this scoping review were to (1) outline the clinical features of shoulder girdle neoplasm that would help primary contact clinicians recognise and refer onwards and (2) quantify the misdiagnosis rate of shoulder girdle neoplasm as FSCS that exists in the literature.

## Materials and methods

This scoping review was structured in line with the Preferred Reporting Items for Systematic Reviews and Meta-analysis Extension for Scoping Reviews (PRISMA-ScR) guidelines.^21^ Four electronic databases (Embase, Medline, PUBMED and Scopus) were systematically searched in March of 2024 using a search strategy consisting of the following key terms: (shoulder OR proximal humerus OR scapula OR clavicle) AND (tumour OR neoplasm OR cancer) AND (frozen OR adhesive OR stiffness). Eligible records were exported to EndNote 20.^22^ Inclusion criteria were (1) full-text cohort studies including shoulder girdle neoplasm with or without a cohort of FSCS and (2) including the details of either the clinical examination or reason for referral.

Critical appraisal of methodological quality was performed using the JBI Critical Appraisal Tool for case series as this was the most frequent study design of the included studies.^23^ The JBI Critical Appraisal Tool utilises 10 questions to grade the quality of a case series with items being addressed as either ‘yes’, ‘no’, ‘unclear’ or ‘not applicable’. The JBI Critical Appraisal Tool does not have its own scoring system. Given this, each included study was given a converted percentage score based on the responses to the 10 questions. Answering ‘yes’ to a question equated to 1 point, while answering ‘no’ or ‘unclear’ resulted in 0 points. The total score out of 10 was then converted to a percentage.

Data charting and items from the included studies were summarised in table form by design, sample size, type and location of neoplasm, number of neoplasms misdiagnosed as FSCS, associated time delays in and between symptom onset and diagnosis, diagnostic methods, and the signs and symptoms from clinical examination or referral. Clinical examination features for all neoplasms were recorded as a percentage of patients who presented with each symptom divided by the total number of patients for whom clinical/referral details were available. Where misdiagnosis as FSCS occurred symptoms were recorded as a percentage of patients who presented with each symptom divided by the total number of patients who were misdiagnosed. As this was a scoping review, no meta-analysis was conducted.

## Results

**Figure 1.**
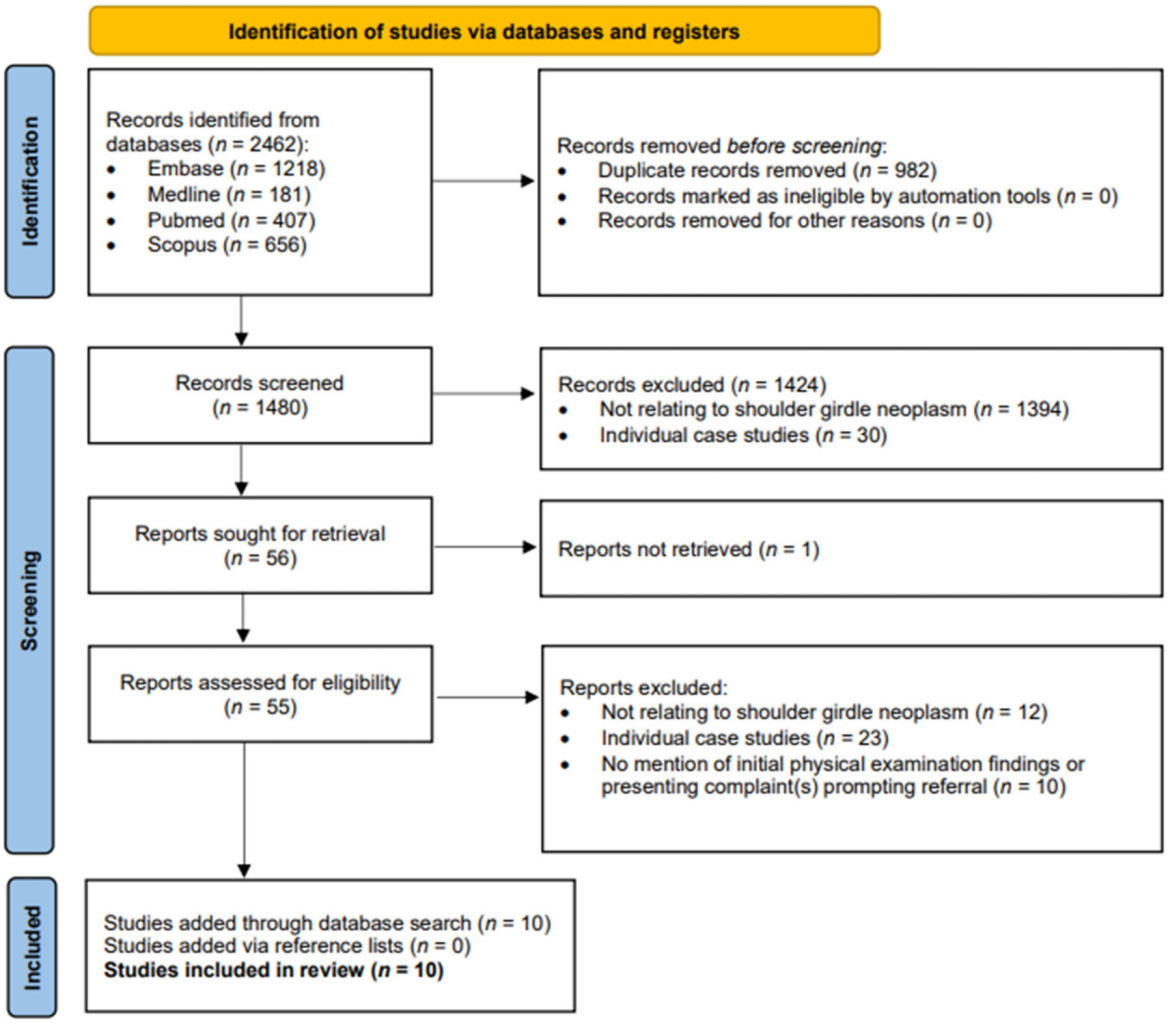
Preferred reporting items for systematic reviews and meta-analyses (PRISMA) flow diagram.

The initial search yielded 2462 results, 982 of which were duplicates (Figure 1). After applying the inclusion/exclusion criteria 56 studies remained and were sought for retrieval. Fifty-five of these studies were retrieved, and 46 were excluded based on our criteria. One study was unable to be retrieved as full text and was excluded.^24^ A total of 10 studies were included in the scoping review. The reference lists of the included studies were examined for further articles that might meet inclusion criteria, but no additional studies were identified in this way.

Of the 10 studies, four were retrospective cohorts^4,20,25,26^ and six were case series.^6,27–31^ JBI methodological grading scores ranged from 50% to 90% (Table 1). Common areas where studies failed to clarify methodology and/or scored poorly on the JBI included questions 4 and 5 regarding consecutive and complete inclusion of participants.

**Table 1.** Study characteristics and methodological grading.

Author (year)	Design	Sample size	JBI grading
Cleeman et al. (2005)	Retrospective cohort	*n* = 194	90%
Glanzmann et al. (2013)	Case series	*n* = 2	80%
Jassim et al. (2020)	Retrospective cohort	*n* = 99	90%
Kapoor et al. (2008)	Case series	*n* = 12	90%
Lin et al. (2014)	Case series	*n* = 5	50%
Mavrogenis et al. (2012)	Case series	*n* = 21	90%
Prabowo et al. (2023)	Case series	*n* = 3	50%
Quan et al. (2003)	Case series	*n* = 5	50%
Robinson et al. (2003)	Retrospective cohort	*n* = 67	90%
Sano et al. (2010)	Retrospective cohort	*n* = 34	70%

A total of 442 patients with shoulder neoplasms were included across the 10 studies. Where bone tumour location was specified, 209 were identified in the proximal humerus, 59 in the scapula and 29 in the clavicle. There were 58 soft tissue neoplasms identified about the shoulder girdle. A specific diagnosis was available for 328 neoplasms and is noted in Table 2. Benign bone tumours were documented most frequently, with 148 occurrences in this review. Malignant bone tumours were the second most common neoplasm, accounting for 122 lesions. Benign and malignant soft tissue tumours accounted for 40 and 18 cases, respectively.

**Table 2.** Type and number of reported shoulder neoplasms.

Type	Number	Type	Number
**Benign bone tumours**		**Benign soft tissue tumours**	
Enchondroma	81	Lipoma	22
Osteochondroma	15	Desmoid	6
Unicameral bone cyst	10	Hemangioma	5
Fibrous dysplasia	7	Myositis ossificans	2
Osteoid osteoma	7	Angiolipoma	1
Langerhans’ cell histiocytosis	5	Ganglion	1
Osteoblastoma	4	Neurofibromatosis	1
Chondroblastoma	3	Pigmented villonodular synovitis	1
Giant cell tumour	3	Synovial chondromatosis	1
Eosinophilic granuloma	2		
Perichondroma	2	**Malignant soft tissue tumours**	
Condensing osteitis	1	Metastatic soft tissue tumour	7
Ganglion	1	Dermatofibrosarcoma protuberans	2
Hemangioendothelioma	1	Plasmacytoma	2
Nonossifying fibroma	1	Angiosarcoma	1
Periosteal desmoid	1	Extraosseous chondrosarcoma	1
		Leiomyosarcoma	1
**Malignant bone tumours**		Malignant fibrohistocytoma	1
Chondrosarcoma	53	Primitive neuroectodermal tumour	1
Ewing's sarcoma	11		
Osteosarcoma	11		
Malignant fibrohistiocytoma	6		
Multiple myeloma	6		
Lymphoma	5		
Spindle cell sarcoma	1		

Five studies reported patients with shoulder girdle neoplasm that were initially misdiagnosed as FSCS (Table 3).^4,6,20,25,27^ Five studies reported the time from initial symptom onset to final correct diagnosis of neoplasm^4,6,20,27,31^ with one specifically accounting for diagnostic delay due to initial FSCS misdiagnosis.^4^ All but one study reported their diagnostic pathway to final diagnosis^30^ with only one not utilising X-ray.^29^

**Table 3.** Diagnostic details of included studies.

Author (year)	Misdiagnosed as FSCS	Average time to diagnosis (months)	Reported diagnostic methods
Cleeman et al. (2005)	–	–	Radiology (unspecified), histopathology
Glanzmann et al. (2013)	50% (*n* = 1)	30	X-ray, CT, MRI
Jassim et al. (2020)	9% (*n* = 8)	–	X-ray, CT, MRI, histopathology
Kapoor et al. (2008)	–	–	X-ray, CT, MRI, bone scan
Lin et al. (2014)	–	–	CT, MRI
Mavrogenis et al. (2012)	–	–	–
Prabowo et al. (2023)	–	11	X-ray, CT, MRI
Quan et al. (2003)	100% (*n* = 5)^a^	19	X-ray, CT, MRI, bone scan, histopathology
Robinson et al. (2003)	10% (*n* = 7)	16	X-ray, CT, MRI, bone scan
Sano et al. (2010)	26% (*n* = 9)	9	X-ray, CT, MRI, histopathology

FSCS: frozen shoulder contracture syndrome; CT: computed tomography; MRI: magnetic resonance imaging.

^a^ Study only presented cases of shoulder neoplasm misdiagnosed as FSCS.

**Figure 2.**
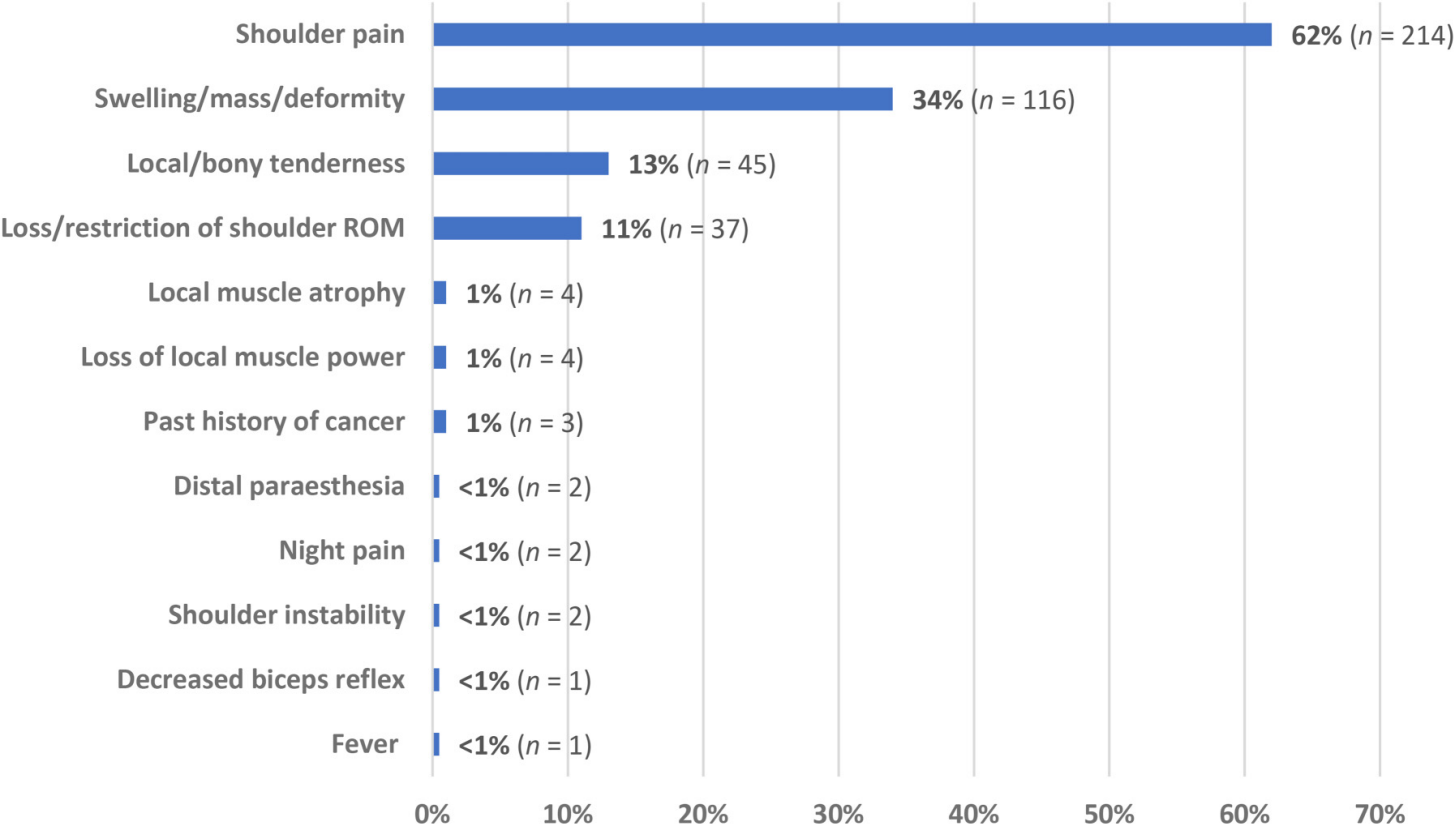
Complaints and examination findings in patients with shoulder girdle neoplasms (*n *= 346).

Clinical examination findings for participants diagnosed with a shoulder girdle neoplasm were reported in 346 patients across the 10 studies (Figure 2). The most reported complaint in patients with shoulder neoplasm was shoulder pain (62% of cases), followed by the presence of a localised shoulder girdle mass, swelling, and/or deformity (34% of cases). Local tenderness and loss/restriction of shoulder ROM and accounted for 13% and 11% of patient symptoms, respectively. All other clinical and/or historical findings accounted for ≤ 1% of all reported presentations.

**Figure 3.**
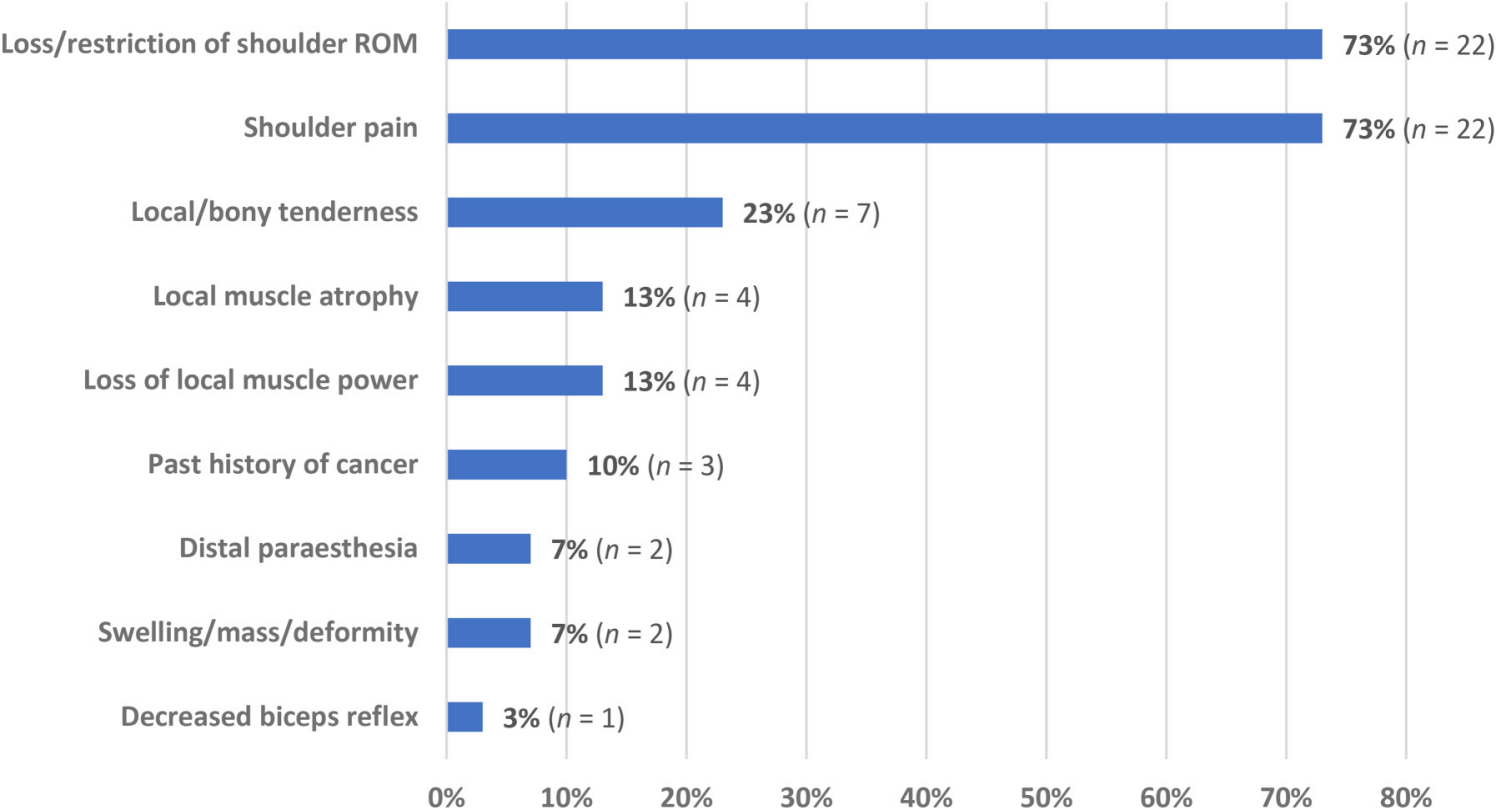
Complaints and examination findings in patients with shoulder girdle neoplasm misdiagnosed as frozen shoulder contracture syndrome (FSCS) (*n *= 30).

Clinical findings for cases with shoulder neoplasm who were initially misdiagnosed as having FSCS were reported in 30 participants (Figure 3). The most frequently reported symptoms in this group were loss/restriction of shoulder ROM and shoulder pain (73% each), followed by local/bony tenderness on palpation (23%) and local muscular atrophy and loss of power (13%).

## Discussion

This scoping review aimed to outline the clinical features of shoulder girdle neoplasm and document the rate and clinical features of misdiagnosis as FSCS. We identified 10 studies that reported the clinical features of shoulder girdle neoplasm and of these five reported specific cases and clinical features of misdiagnosis as FSCS. Across the 10 studies ∼15% (30/442) of participants were initially diagnosed as FSCS but later confirmed as shoulder girdle neoplasm, with the reported rate of misdiagnosis ranging between 9%^25^ and 50%.^27^

The overall incidence of patients with shoulder pain and ROM loss presenting with local neoplasm is relatively low, accounting for ≤ 1% of patient presentations.^4^ However, in this small percentage of patients, misdiagnosis can lead to delays and/or inappropriate treatment. One retrospective cohort reported that FSCS misdiagnosis led to a significant delay in the identification of malignant tumours (9 months) when compared to those without misdiagnosis (3 months) (*p *= 0.035). Even in studies where there was no FSCS misdiagnosis, the time to final diagnosis of neoplasm ranged from 11 months^31^ to 30 months.^27^ In each of these studies it was not well delineated whether these timeframes were due to delays in patient presentation from the time of symptom onset, lack of follow-up, or waiting times for imaging or histopathology. A total of 27% of patients with a misdiagnosed shoulder neoplasm (*n *= 9) underwent subsequent inappropriate and contraindicated procedures. This manifested as four cases of hydrodilatation, four cases of arthroscopic surgery and one manipulation under anaesthesia.^6,32^ These inappropriate interventions add another layer of potential harm due to misdiagnosis outside of diagnostic delay due to increasing the potential risk of neoplastic spread.

The most prevalent clinical feature of shoulder girdle neoplasm was localised shoulder pain, present in nearly two-thirds (62%) of all reported cases. The presence of a localised mass, swelling, or deformity was the second most common complaint (34%), followed by local and/or bony tenderness (13%). Loss of glenohumeral ROM was the fourth most common clinical feature, reported in 11% of all cases; however, most studies in this review failed to differentiate whether ROM loss was active or passive, self-reported or clinician-measured, and/or the extent of the ROM impairment. The partial exceptions to this were two retrospective cohorts which classified FSCS patients as having < 120° of glenohumeral elevation^4^ or < 90° of abduction and < 20° of external rotation.^20^ FSCS should be suspected when there is an equal loss of both active and passive abduction and external rotation with largely normal glenohumeral X-rays.^33,34^ With the absence of this specific criteria being met and/or reported in the current studies, it is difficult to determine if misdiagnosis rates in several studies may be related to the criteria used to define impaired glenohumeral ROM in cases of suspected FSCS. As such, both active and passive ROM loss should be quantified when suspecting FSCS.^33,34^ Furthermore, the description of ‘shoulder stiffness’ was used in several studies to describe both subjective patient complaints and physical examination findings. From a biomechanical perspective, joint stiffness describes the relationship between the amount of force required to passively move a joint.^35,36^ While previous research has attempted to quantify patient-reported descriptions such as ‘tightness’,^37^ the specific relationship between subjective and objective measured of joint stiffness is not well quantified in FSCS, shoulder neoplasm, or any other pathology where loss of ROM is part of the clinical presentation. As such, while ‘stiffness’ as described subjectively by a patient may help the clinician broadly categorise differential diagnosis, it may not be an accurate clinical or objective descriptor for loss of glenohumeral ROM, passive, or otherwise. As such, the language for documentation around the loss of glenohumeral passive ROM should differentiate between subjective and objective examination findings.

In patients who received a shoulder girdle neoplasm misdiagnosis, loss of glenohumeral joint ROM was the most common complaint along with shoulder pain, with both findings present in 73% of all reported cases. Common clinical features of shoulder girdle neoplasm such as atraumatic swelling and local or bony tenderness were less common in this group accounting for 7% and 23% of patients, respectively, potentially contributing to misdiagnosis in the presence of glenohumeral joint PROM loss. The presence of any undifferentiated atraumatic swelling, mass, and/or deformity is commonly associated with sarcomas and other soft tissue tumours, and should prompt further medical investigations to further differentiate.^38^ Additionally, given that swelling, mass or deformity are not commonly present in FSCS, it should prompt a higher degree of clinical suspicion for neoplastic process. Regional bony tenderness about the acromioclavicular joint has been associated with a significantly increased likelihood of shoulder neoplasm when compared to patients without (*p *≤ 0.01).^20^ Additionally, local bony tenderness has been documented as a common feature for several highly malignant bone cancers including osteosarcoma, chondrosarcoma and Ewing's sarcoma.^39^ Specifically, scapular tenderness has been shown to significantly increase the risk of shoulder lesion malignancy (*p *= 0.001, OR = 8.16, 95% CI 2.86–25.02).^26^ This association between bony scapular tenderness and malignancy is increased when paired with a decreased range of motion about the affected joint,^39^ and may serve as another sign of neoplasm in the absence of swelling, mass and/or deformity. Given these findings, careful observation of the shoulder girdle for swelling, mass or deformity along with purposeful palpation of the clavicle, proximal humerus and scapula should be a standard part of the clinical examination for patients presenting with shoulder pain and loss of PROM.

Red flag symptoms and medical history and such as previous cancers, night pain, and fever were present in ≤ 1% of patients with shoulder neoplasm in this review. In contrast, 10% of patients with a misdiagnosed shoulder neoplasm had a history of cancer noted in their clinical history. However, it is not clear if a history of cancer was elicited at the time of initial misdiagnosis, or at the time of neoplastic diagnosis. The larger retrospective cohorts from this review failed to document the history of cancer or other red flags in their inclusion criteria. As such, the utility of historical and clinical red flags in diagnosing shoulder girdle neoplasm may be understated in this population.

While debate exists regarding the utility of X-ray in differentiating shoulder girdle neoplasm and FSCS, seven of the 10 studies in this review explicitly used plain radiography at some stage along their diagnostic pathway.^4,6,20,25,27,28,31^ Within these studies, undifferentiated lesions were identified on X-ray and used to progress to more specialist imaging (CT, MRI or bone scan) or histology for final diagnosis. Two studies reported that repeated X-rays were used to detect lesions in recalcitrant cases of pain where no bony lesion was initially detected.^4,6^ One cohort reported that all cases of neoplasm misdiagnosis as FSCS were due to normal shoulder X-rays and no palpable mass on physical examination.^20^ This accounted for 10% (*n *= 7) of their cohort, each of which were bone tumours. The remaining 90% were able to be identified using X-ray and/or clinical examination. Larger cohort studies have documented the diagnostic accuracy of X-rays in detecting skeletal malignancy between 69.5% and 87.0% when used alongside bone biopsy as the gold standard.^40,41^ The largest of these cohorts reported X-rays as having high specificity (96.1%) but low sensitivity (33.0%) in detecting malignant bone lesions.^40^ In contrast, the role of X-ray in the detection of soft tissue sarcoma is limited to when lesions present with calcification which may only characterise specific variants.^42,43^ As such, the preferred initial imaging pathway for suspected soft tissue neoplasm is ultrasound which has reported sensitivity and specificity of 84.0% for the detection of malignant lesions.^43^ Given this, physical examination features such as local/bony tenderness indicative of bone cancers may direct the primary contact clinician towards X-ray investigation at minimum, while local swelling, mass or deformity may be directed towards ultrasound at minimum under suspicion of soft tissue neoplasm.

**Figure 4.**
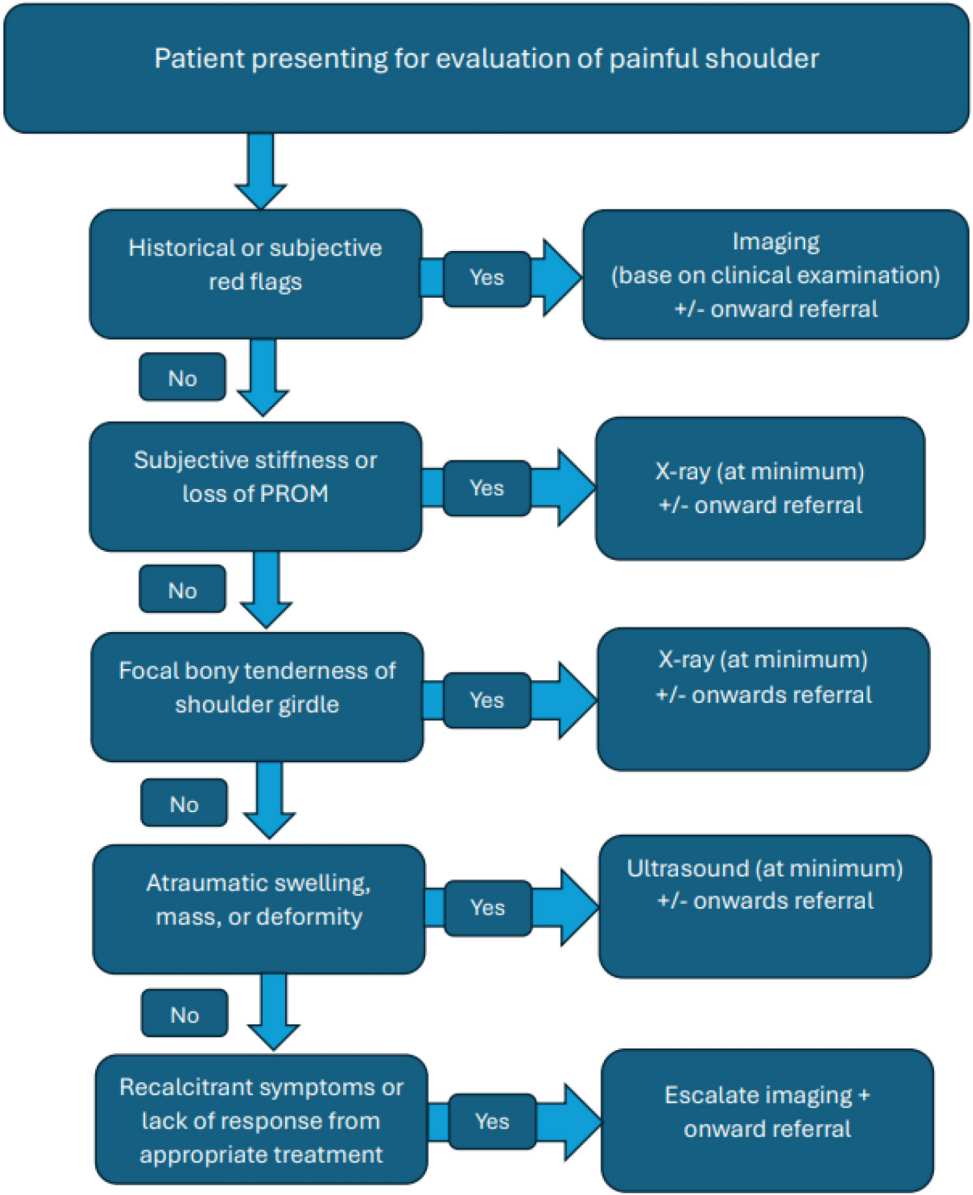
Proposed flowchart for patients presenting to primary contact clinicians with shoulder pain.

Most patients from this review with a diagnosis of shoulder girdle neoplasm presented with shoulder pain and some combination of subjective stiffness/loss of ROM, local/bony tenderness and/or atraumatic swelling, mass or deformity. Given this, we have constructed a flowchart to help guide the decision making of primary contact clinicians (Figure 4). We have suggested the use of X-ray (at minimum) in the presence of reduced glenohumeral joint PROM given its use in differentiating many other pathologies that may cause a reduction in glenohumeral PROM. Additionally, of the diagnosed shoulder neoplasms in this review, 72% were bony neoplasms (*n *= 237) which would have a higher likelihood of being diagnosed with X-ray. When applying this flowchart to patients from studies included in this review, it was able to raise suspicion of neoplasm in 96% of cases where individual patient complaints were available from the authors (*n *= 252). The remaining 4% of patients (*n *= 11) were asymptomatic for pain or key features of neoplasm identified in the flowchart. Given the lack of a standardised physical examination or consistent definition of the term stiffness used by the included studies, further evaluation of this tool is recommended to determine its validity.

The main limitations of this review relate to the methodology and number of the studies included. As all studies in this scoping review were retrospective cohorts or case series, and there were relatively few studies to draw upon for the review it limits the strengths of our conclusions. Given the broad heterogeneity of outcome measures and statistical methods, no meta-analysis was able to be conducted.

## Conclusion

While the incidence of shoulder girdle neoplasm in the general population is low, many patients with shoulder neoplasm will experience diagnostic delay and/or inappropriate treatment due to misdiagnosis as FSCS. The presence of localised swelling, mass or deformity and/or discrete bony tenderness can aid the primary contact clinician in differentiating the oncological process from FCSC when loss of ROM is present and should be included in a routine examination. Traditional red flag symptoms of cancer such as the history of oncology, fever and night pain were not frequently documented in this scoping review, though may be underreported in the literature on shoulder neoplasm. This review noted X-ray is often utilised to begin the diagnostic pathway in most patients with shoulder neoplasm. However, X-rays are only likely to have utility when bone lesions are the primary differential diagnosis. As such, clinical examination findings should help guide the initial imaging strategy.

## Highlights

- Patients with shoulder girdle neoplasm are at risk for misdiagnosis given the overlap in age and symptoms with frozen shoulder contracture syndrome.
- The difference between subjective stiffness and active or passive ROM deficits is poorly defined and inconsistently reported in patients with shoulder neoplasm.
- In patients with glenohumeral joint ROM loss, discrete palpation for localised bony tenderness of the shoulder girdle may help differentiate between pathologies.
- The choice of initial imaging for patients with glenohumeral joint ROM loss should be based on clinical examination findings such as bony tenderness and local swelling, mass, or deformity.
